# Enteric Elongation Induced by a Novel Sleeve Device in a Live Roux-en-Y Configuration

**DOI:** 10.3390/bioengineering12070771

**Published:** 2025-07-17

**Authors:** Joshua C. Colvin, Collyn C. O’Quin, Hannah R. Meyer, Valerie L. Welch, Giovanni F. Solitro, Jonathan S. Alexander, Donald L. Sorrells

**Affiliations:** 1 Department of Surgery, LSU Health Shreveport, Shreveport, LA 71103, USA; 2 Department of Pathology, LSU Health Shreveport, Shreveport, LA 71103, USA; 3 Department of Orthopedic Surgery, LSU Health Shreveport, Shreveport, LA 71103, USA; 4 Department of Molecular and Cellular Physiology, LSU Health Shreveport, Shreveport, LA 71103, USA; jonathan.alexander@lsuhs.edu

**Keywords:** Short Bowel Syndrome, distraction enterogenesis, roux limb, medical device

## Abstract

Short bowel syndrome (SBS) is characterized by insufficient intestinal length to support absorption causing malnutrition. The bowel adapts to SBS via intestinal dilation and delayed gastric emptying but still often requires long-term parenteral nutrition. Current surgical options to lengthen the bowel pose significant risks and often provide limited expansion. ‘Distraction enterogenesis’ has been proposed as a technique to induce intestinal lengthening for SBS. The deployment of the intestinal expansion sleeve (IES) device is hypothesized to result in significant intestinal lengthening in vivo. A Roux-en-Y was created in the jejunum of seven rats for isolated IES deployment. The IES was precontracted over a Bucatini noodle and inserted into the isolated roux limb. After 4 weeks of deployment, rats were sacrificed, Roux-en-Y length recorded, and histology analyzed. A paired *t*-test was performed to compare initial and final roux limb lengths and histopathological tissue remodeling. Intestinal distraction evaluated at 4 weeks post deployment of the IES resulted in a significant 30.2% elongation in roux limb length (43.6 ± 14.4 mm to 56.4 ± 20.8 mm (*p* = 0.043, n = 7). IES samples showed changes in mucosal and submucosal integrity and bowel wall thickness in response to IES lengthening. In samples with partial mucosal erosion, the basal/regenerative layers of the mucosa were preserved. Distraction enterogenesis with significant intestinal lengthening in vivo has been achieved with the IES device. Histologic changes suggest all bowel functional layers and attributes are maintained through distraction enterogenesis. Future constructs of the IES may benefit from the addition of immunomodulators. Increasing intestinal mass with these devices may complement the treatment paradigm for SBS.

## 1. Introduction

Short bowel syndrome (SBS) is characterized by insufficient intestinal length leading to malabsorption and malnutrition. The partial surgical resection of the small bowel is the most common cause of SBS, but it can also be caused by any disease or injury that prevents the proper function of the bowel [1,2]. Acquired SBS is more common than congenital forms [3]. In pediatrics, SBS is most commonly due to necrotizing enterocolitis, malrotation with volvulus, or intestinal malformations including omphalocele and gastroschisis [2,4,5]. The overall incidence of pediatric SBS is 22.1 cases per 1000 admissions to the neonatal intensive care unit (NICU) and 24.5 cases per 100,000 live births [6].

The normal adult small intestine length is around 600 cm (19.7 ft) and normal colon length is 150 cm (4.9 ft). Total GI tract length is 750 cm (approximately 25 ft); however, in SBS patients, the average GI tract length is only 6.5 ft [3]. Clinical symptoms arise when the remaining gastrointestinal tract is unable to compensate for the lost length; therefore, SBS largely reflects the loss of functional absorptive capacity [7]. SBS symptoms vary depending on the extent and location of missing bowel sections, and diarrhea is common, causing dehydration, weight loss, and malnutrition [8]. Malnourished patients often exhibit the swelling of the abdomen, muscle wasting, and deficiencies in vitamins (K, B1, and B12) and minerals (potassium, calcium, magnesium, and zinc), causing a broad constellation of symptoms [3,8].

The less invasive management for SBS includes long-term total parenteral nutrition (TPN) support and pharmacological management to improve intestinal function and/or slow the transit time through the intestines [1,4,9]. SBS patients adapt naturally by delayed gastric emptying and intestinal dilation, but this can take years and often requires parenteral nutrition for adequate growth [10]. Surgical options to increase bowel length have been performed mostly in the pediatric population as opposed to adults, and these procedures may pose significant risks and often provide limited expansion [7]. These methods range from intestinal transplants to various surgical elongation procedures such as the Bianchi procedure and serial transverse enteroplasty (STEP) procedure [7,11,12,13]. The Bianchi procedure, otherwise known as the longitudinal intestinal lengthening and tailoring (LILT) procedure, consists of longitudinally dividing the bowel in half and then stapling the bowel [5,11]. After creating the two sections of bowel with a smaller diameter, the ends are sewn together, doubling the length of bowel [2,11]. In order for the Bianchi procedure to be successful, the initial bowel needs a minimal length of 20–40 cm, and the diameter must be dilated to at least 200% [2]. The STEP procedure is performed by stapling the bowel transversely across half of the luminal diameter and alternating sides, lengthening the bowel and decreasing the diameter [1,12]. However, an important aspect to consider with these procedures is that it can take years to dilate the bowel to a diameter required to perform either of these operations [14]. The earlier the intestinal elongation procedures are performed, the less TPN is needed for these patients [4].

Distraction-induced enterogenesis, a form of controlled tissue expansion, has been shown to produce significant bowel lengthening with the proliferation of the epithelial structure allowing the preservation of intestinal function [15,16,17,18]. This was performed in an excised length of a bowel that was re-implanted back into the rat model, showing that the re-implanted bowel still grows and functions like the native bowel [15]. Spencer et al. used a hydraulic-driven dual concentric piston made with telescoping syringes, which were inserted into the lumen of the isolated bowel in pigs. After device stabilization, the pigs were closed up to allow for one week of recovery before device activation to elongate the bowel, resulting in significantly longer bowel segments compared to the control segments. These lengthened segments had an almost 2-fold increase in surface area [19]. In rats, Safford et al. used an intestinal lengthening device that was inserted into the lumen of the bowel and secured to the external oblique fascia. After recovery, the device was manually lengthened by 1 mm per day. Once the device was removed, the intestine was 114% longer in length and had increased function [20].

We performed an ex vivo investigation using small intestines from New Zealand White rabbits, where our IES device showed a 36.2 ± 11% increase in small bowel length; however, intestinal wall thinning was also noted [10]. Similarly, our previous ex vivo mechanical testing proof of concept study in Sprague Dawley rat small intestines showed that the force required to rupture the small bowel was 1.88 ± 0.21 N, while the IES device exerted 0.22 ± 0.01 N, only 11.7% of the tension that produced failure. The deployment of the IES device ex vivo resulted in a 21 ± 8% increase in rat small bowel length. However, as before, this produced mucosal loss and tissue thinning [21]. Our studies show that our IES device is capable of lengthening the intestinal tissue without failure (i.e., providing longitudinal expansion while preserving the intestinal architecture). In vivo, the ‘distractive’ forces of the IES device should allow the small bowel mucosa to accommodate and remodel while stretching, thereby lengthening the bowel while avoiding the persistent thinning of the intestinal walls. IES devices could be used to treat both pediatric patients and those with acquired SBS by restoring small bowel length through ‘distraction enterogenesis’. Our hypothesis is that deployed IES devices will result in significant intestinal lengthening in vivo while preserving normal intestinal architecture.

## 2. Materials and Methods

**Figure 1:**
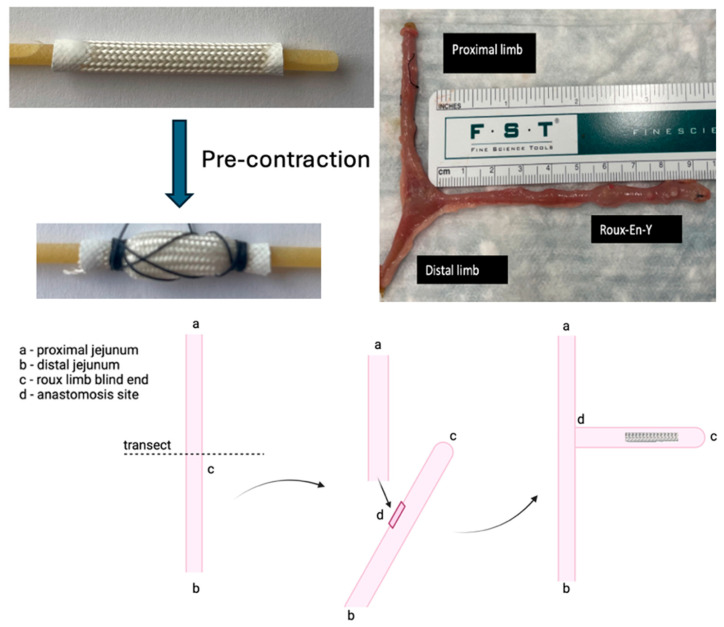
Precontraction of IES device, and creation of Roux-en-Y.

The intestinal expansion sleeve (IES) is a 30 mm cylindrical material with helicoid trusses. Each end was painted with a rubber sealant to prevent the fraying of the device while contracted over a bucatini noodle, a rigid polymer support. The device was precontracted and fixated using absorbable suture to allow the device to be deployed into the intestine and re-expand once the absorbable suture and bucatini noodle support dissolved (Figure 1). Isofluorane anesthesia was used per nose cone to the level of unresponsiveness to the pinch test. Carprofen was administered intraoperatively subcutaneously in a standard dose for postoperative pain control.

In seven Sprague-Dawley rats, a Roux-en-Y was constructed with 5-0 silk and measured in vivo along the mesenteric border with a ruler while lax and with no tension applied to the intestinal tissue. (Figure 1). After the creation of the roux limb, the precontracted IES device was inserted into the isolated intestinal limb and fixated to the intestinal wall using a 4-0 silk permanent suture at the ends. The absorbable suture holding the device in a precontracted state and the polymer support dissolved, allowing the device to re-expand. As the device re-expanded, the permanent suture maintained placement in the small bowel, and the force promoted ‘distraction enterogenesis’.

The first group of controls, consisting of two rats, underwent surgery to create a Roux-en-Y of the small bowel, but no IES device was inserted. The other control group, consisting of two rats, had a Roux-en-Y performed and a non-contracted IES device inserted and stabilized. All rat groups were allowed 4 weeks of daily observation and weighing before the final measurement of the roux limb. The roux limb and anastomosis were isolated, measured in vivo while lax and under no tension, and resected. IES devices were removed from the roux limb by cutting the sutures, and the rigid polymer support had already been degraded by intestinal enzymes at the time of device removal. Roux limbs were then placed in 3.7% phosphate-buffered formaldehyde for 24 h to be processed by the LSU Pathology Core laboratory. Following fixation and processing, tissue was embedded in paraffin for sectioning. Sections 5 µm thick were cut and stained with hematoxylin/eosin for analysis.

## 3. Results

### Intestinal Lengthening

**Figure 2:**
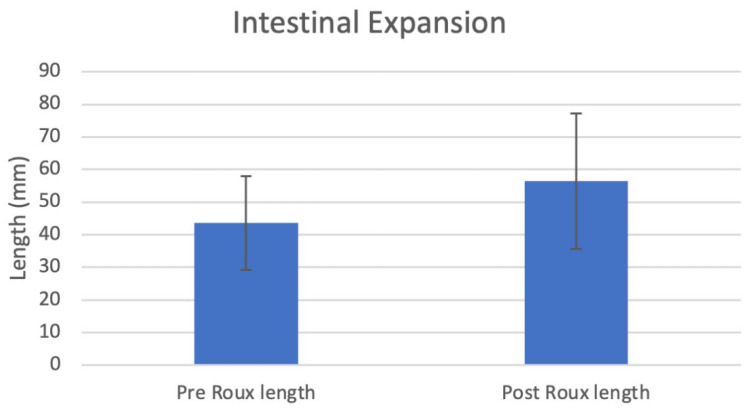
Intestinal length pre and post expansion.

The deployment of the IES device in rat small intestines over one month produced a lengthening of 43.6 ± 14.4 mm to 56.4 ± 20.8 mm (*p* = 0.043, n = 7), a 30.2% elongation (Figure 2, Table 1).

The two control rats with roux limbs and no IES device had an initial roux length of 40 mm, which remained at 40 mm; similarly, the two controls with roux limbs with unexpanded IES devices had an initial roux length of 60 mm, which remained at 60 mm of length.

**Figure 3:**
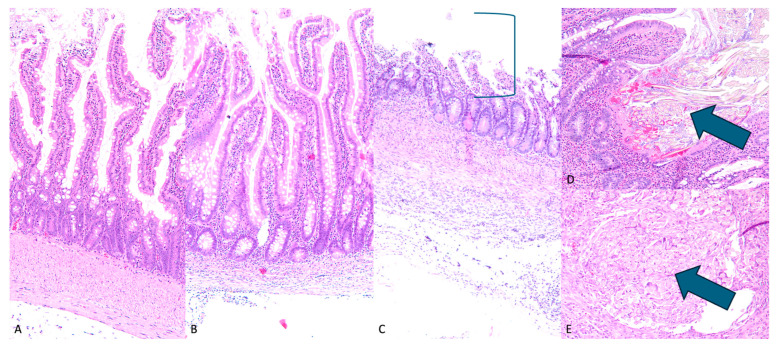
H&E intestinal histology. (**A**) (100×)—control Roux-en-Y with unremarkable intact small bowel, (**B**) (100×)—IES lengthened bowel with intact mucosal villi length and integrity, (**C**) (100×)—IES induced partial erosion of mucosa (mucosal erosion shown by bracket), with preserved basal/regenerative layer, (**D**) (100×)—intact intestinal mucosa with associated suture material (indicated by arrow), (**E**) (100×)—submucosal suture granuloma (indicated by arrow).

A histologic analysis of the non-contracted and pre-contracted IES-exposed intestinal tissue revealed predominantly intact surface mucosa and villous architecture with foci of partially eroded surface mucosa, while for the control Roux-en-Y with no-IES group, intestinal tissue architecture was completely preserved and unremarkable. Of note, suture material was identified in multiple sections of both control and IES-exposed intestinal tissue, consistent with post-operative surgical changes. Distinct suture granulomas with varying degrees of surrounding foreign body granulomatous inflammatory reaction were seen in both control tissues and in IES-exposed tissues. Additionally, the bowel wall of IES-exposed tissue contained mild scattered chronic inflammation, independent of the suture granulomas. As shown in Figure 3, the bowel wall thickness was variable among control and IES-exposed tissue, with no distinct trend in thickness based on the presence or absence of an IES device.

## 4. Discussion

Short bowel syndrome affects 24.5 out of every 100,000 live births and has diverse symptoms, depending on the degree of shortening and the anatomical location of the bowel that is affected [3,6]. Multiple treatment options exist for SBS, ranging from intestinal adaptation with TPN support to surgical management [4]. Physiologic response that improve intestinal adaptation (remodeling) increase bowel diameter and promote gastroparesis, allowing a slower transit time for nutrient absorption [2,13,22]. However, such adaptation can take years to occur, delaying most surgical procedures for SBS [14,19].

Our previous ex vivo study demonstrated that IES devices provide adequate lengthening with a distractive force well below the failure load of the rat small bowel and are safe to use in vivo without occurrences of small bowel rupture/perforation [21]. In this in vivo study, we successfully showed that the deployment of our IES device can significantly lengthen the small bowel, allowing ‘distraction enterogenesis’ to take place, which preserves normal intestinal architecture following the lengthening procedure.

After a one-month deployment of our IES device in rats, the average intestinal length increased from 43.6 ± 14.4 mm to 56.4 ± 20.8 mm (*p* = 0.043, n = 7), a 30.2% elongation. All rats survived the entire month of post-operative monitoring, with no anastomotic leaks or other surgical complications. Two of the rats had a notable small decrease in the intestinal length of the roux limb post deployment, which increased the variability and decreased the statistical power of the study. One of the rats had device maldeployment with only one side of the sutures remaining intact throughout the lengthening process, while the other rat was completely missing the IES device upon examination. Due to the nature of the surgical procedure and device, the device location is unable to be seen after the procedure until the retrieval of the roux limb and device, so it was unable to be determined exactly when the devices stopped functioning as intended. These device complications could explain the failure of the roux limb lengthening.

Currently, this study is testing the IES device in a blind-ended roux limb, out of line with the intestinal flow, requiring invasive surgery. The use of the roux limb allows for mechanical testing but removes the area of interest out of normal intestinal flow and physiologic stressors that could affect the device’s effectiveness and response of the intestinal tissue to the device. The rats in this study did not have short bowel syndrome. The deployment of these expanders in rats with short bowel syndrome was obviously not performed. The physiologic response of rats with short bowel syndrome might be different from the rats (with normal bowel length) that we used. However, this is a proof of concept that demonstrates an initial ability to place the expander and obtain the apparent expansion of the intestine. The in-line deployment of the IES device is anticipated to allow the insertion of the device without a need to operate. As IESs are contracted, the diameter increases, allowing for better fixation to the intestinal walls. When they re-expand, the diameter correspondingly decreases. Our goal is to ultimately deploy the precontracted IES device by a long feeding tube, allowing delivery and fixation to the intestinal wall. Therefore, as the device re-expands to lengthen the intestine, it will decrease in diameter, release itself from the intestinal wall, and be passed out through the gastrointestinal canal. Feeding tube deployment could offer the possibility of an intermediate management when TPN and intestinal adaptation are not enough while avoiding invasive surgeries like intestinal transplants, a Bianchi procedure, and an STEP procedure that require general anesthesia and hospitalization. Additionally, treatment with the IES device can be performed before gradual intestinal adaptation occurs, unlike the Bianchi procedure and STEP procedures [11,12].

This histologic review reveals normal small bowel histology with preserved surface mucosa and villous architecture. Although there are some foci of partial mucosal erosion in both groups of IES-exposed tissue, this appears to be likely secondary to physical contact with the IES device and not as a result of ‘distraction enterogenesis’. Notably, the basal portion of the mucosa is preserved; therefore, the small bowel does retain its ability for self-repair following the removal of the device. Suture material, distinct submucosal suture granulomas, and associated submucosal granulomatous reaction were observed; however, these are not significantly different compared to control tissues with IES exposure. Given the final lengths following stretching with the IES device, the suture and associated inflammatory responses do not seem to compromise the ability of the bowel wall to lengthen. The chronic inflammation and apparent thinning seen in the bowel wall away from the granulomatous suture reaction are likely secondary to bowel wall stretching and do not appear to irreversibly affect the tissue structure. The histologic findings are encouraging and suggest that the use of the IES device does not cause severe or permanent damage to the bowel wall. Therefore, the structure and function of the bowel are maintained following IES device exposure.

The post-treatment evaluation of expanded intestinal function was not performed and is a limitation of this study. The maintenance of new intestinal growth cannot be assessed by this study. Histologically, in places with mucosal erosion, the basal/regenerative layer remains intact, suggesting that the mucosa can self-repair and retain normal function. Longer observation times would be needed to allow for the mucosa to regenerate to determine if the superficial mucosal layers and bowel wall thickness would return to normal. Future studies with longer recovery times after device deployment and removal are planned to analyze the long-term effect of the IES device on the bowel mucosa to establish if the increased length is retained or if intestinal length regression occurs after device removal. While we do not have post-treatment assessments on intestinal function in this study, all of the rats maintained and gained weight during the study period, indicating that the nutritional function of the bowel is maintained. Additional studies for intestinal function could include an measurement of intestinal mass and dextran permeability studies. This study demonstrated the structural gain-of-length benefits of the IES device; however, the long-term and functional effects of the increased length have yet to be established.

Another limitation of the study is that only a single IES device was deployed per procedure. Each SBS patient requires different distraction lengths to become independent of TPN. A study performed by Dubrovsky et al. describes that a bowel length (cm) to body weight (kg) ratio of 1.0 cm/kg is necessary for adult SBS patients to become independent of TPN [23]. In future studies, multiple rounds of IES deployment will likely be needed to develop adequate lengthening to treat SBS.

In this study the initial Roux-en-Y lengths were not all equal, ranging from lengths of 20 mm to 60 mm, largely due to the development of this new surgical technique to make the blind-ended roux limb for device placement. This made the standard deviation of the pre-IES and post-IES deployment lengths larger than what we anticipated. In future studies, we will have Roux-en-Y lengths set to a fixed standard to better determine the lengthening capacity of the 3 cm IES device. Also, the measurement of the Roux-en-Y lengths is subject to human error. Due to the nature of this device, a decrease in length is not expected unless a measurement error occurs or the IES device fails to deploy. Using this rat model, we are unable to fully gauge the discomfort of the IES device. However, as all animals maintained their weight and daily activity with no agitation during handling, we can conclude that they do not display overt pain or distress [24].

## 5. Conclusions

Our study shows that significant bowel lengthening is achieved through ‘distraction enterogenesis’ using our novel IES device. Histopathological analysis revealed no significant differences in gut architecture between control and IES device-treated bowels. Future testing multiple IES devices and therapeutic impregnated IES devices could improve when and how IES are applied as important treatments for patients with SBS.

## Figures and Tables

**Table 1:** Intestinal length pre and post expansion.

	Pre Roux Length (mm)	Post Roux Length (mm)	Roux Limb Elongation (%)	Compressed IES Length (mm)	IES Length (mm)	Initial Weight (g)	Final Weight (g)
IES 1	35	46	31.43	15	30	741	781
IES 2	20	34	70	20	30	659	652
IES 3	40	39	−2.5	20	30	557	566
IES 4	40	53	32.5	20	30	731	741
IES 5	60	58	−3.33	15	30	590	593
IES 6	60	95	58.33	17	30	617	610
IES 7	50	70	40	19	30	788	780

## Data Availability

The data presented in this study are available on request from the corresponding author.

## Abbreviations

The following abbreviations are used in this manuscript:

SBS	Short Bowel Syndrome
IES	Intestinal Expansion Sleeve
TPN	Total Parenteral Nutrition
STEP	Serial Transverse Enteroplasty Procedure
LILT	Longitudinal Intestinal Lengthening
